# Design and Analysis of an Efficient Energy Algorithm in Wireless Social Sensor Networks

**DOI:** 10.3390/s17102166

**Published:** 2017-09-21

**Authors:** Naixue Xiong, Longzhen Zhang, Wei Zhang, Athanasios V. Vasilakos, Muhammad Imran

**Affiliations:** 1 School of Optical-Electrical and Computer Engineering, University of Shanghai for Science and Technology, Shanghai 200093, China; xiongnaixue@gmail.com (N.X.); zlzlmf@163.com (L.Z.); 2 Department of Mathematics and Computer Science, Northeastern State University, Tahlequah, OK 74464, USA; 3 Department of Computer Science and Technology, Hangzhou Dianzi University, Hangzhou 310037, China; 4 Department of Computer Science, Electrical and Space Engineering, Lulea University of Technology, 97187 Lulea, Sweden; athanasios.vasilakos@ltu.se; 5 College of Computer and Information Sciences, Almuzahmiyah, King Saud University, Riyadh 11451, Saudi Arabia; cimran@ksu.edu.sa

**Keywords:** ad hoc network, mobile communication, synchronous checkpointing, asynchronous checkpointing, hybrid checkpointing

## Abstract

Because mobile ad hoc networks have characteristics such as lack of center nodes, multi-hop routing and changeable topology, the existing checkpoint technologies for normal mobile networks cannot be applied well to mobile ad hoc networks. Considering the multi-frequency hierarchy structure of ad hoc networks, this paper proposes a hybrid checkpointing strategy which combines the techniques of synchronous checkpointing with asynchronous checkpointing, namely the checkpoints of mobile terminals in the same cluster remain synchronous, and the checkpoints in different clusters remain asynchronous. This strategy could not only avoid cascading rollback among the processes in the same cluster, but also avoid too many message transmissions among the processes in different clusters. What is more, it can reduce the communication delay. In order to assure the consistency of the global states, this paper discusses the correctness criteria of hybrid checkpointing, which includes the criteria of checkpoint taking, rollback recovery and indelibility. Based on the designed Intra-Cluster Checkpoint Dependence Graph and Inter-Cluster Checkpoint Dependence Graph, the elimination rules for different kinds of checkpoints are discussed, and the algorithms for the same cluster checkpoints, different cluster checkpoints, and rollback recovery are also given. Experimental results demonstrate the proposed hybrid checkpointing strategy is a preferable trade-off method, which not only synthetically takes all kinds of resource constraints of Ad hoc networks into account, but also outperforms the existing schemes in terms of the dependence to cluster heads, the recovery time compared to the pure synchronous, and the pure asynchronous checkpoint advantage.

## 1. Introduction

### 1.1. Research Motive

As a new kind of network technology in the wireless communication field, the mobile ad hoc network, which doesn’t depend on fixed infrastructures, has been widely applied to different kinds of situations such as offices, industries and military, etc., bcause it can not only construct a multi-hops network with the existing network, but also support wireless transmission of data, voices and images under bad environmental conditions by temporarily constructing a smart network.

However, mobile ad hoc networks have some limitations such as lack of support of fixed networks, limited resources and changeable topological structure. Compared to the normal mobile network, its stability is worse and the failure probability is higher. Thus, it is necessary to research fault tolerance and reliability technologies for the network.

In distributed systems, in order to ensure global consistency, when failures occur, the related processes firstly rollback the executed operations and then redo them. Checkpoint technologies are proposed for avoiding rollback to the initial states of the processes [1,2]. Namely, the processes are required to store their current states at a regular or unregularly period, so they just need to roll back to a specific checkpoint instead of the initial state when a failure occurs, to greatly reduce the recovery time [3,4].

In essence, the mobile ad hoc network is a kind of distributed network, hence the checkpoint technologies for it not only need to consider the features of distributed systems and regular mobile networks such as mobility of location and frequent disconnection, but also need take its own unique characteristics into account [5]:

*Multi-hops routing (MH)*. Without the support of a fixed network, the communication among MH_s_ in different clusters needs to be transmitted by multiple intermediate nodes. This will increase the number of wireless communication messages and communication delays for the checkpointing procedure.

*Lack of support for fixed networks*. In regular mobile networks, the checkpoint information of MH_s_ is mostly stored in the Mobile Support Stations (MSSs) dispatching in the fixed network because of the unreliability of storage in MH_s_. However, there are no longer fixed hosts like MSSs in mobile ad hoc networks, hence, the existing checkpointing methods in the mobile computing environment which are dependent on MSSs are not suitable for the ad hoc network any more.

*Changeable topological structure*. In ad hoc wireless networks, the MH*_s_* usually leave one cluster and enter another cluster, which will easily cause a change of the network topological structure. The checkpoint technologies should be able to handle these location changes to ensure the correctness of recovery when handoff occurs [6,7,8].

Therefore, the existing checkpoint technologies for fixed networks and regular mobile networks cannot be applied to mobile ad hoc networks effectively. It is necessary to design new checkpoint technologies for coping with the upper challenges to improve the fault tolerance and reliability of mobile ad hoc networks.

### 1.2. Related Works

Checkpoint technologies were first proposed for improving the fault tolerance ability of distributed systems. According to the relations between checkpoints, the checkpoint technologies can be divided to synchronous checkpointing [3,9,10,11,12] and asynchronous checkpointing [13,14].

Synchronous checkpointing, also called coordinated checkpointing, is the method where all processes stay coordinated and store their states in some stable storage. This kind of checkpoints requires that the processes taking part in checkpointing do the checkpoints synchronously. Its advantages are a lack of cascade rollback and low recovery overhead, whereas it will require a lot of coordination messages and its checkpoint time is longer. On the basis of the interruption of the basic operations of processes, the synchronous checkpointing strategies could be further classified into two kinds: unblocking [3,9] and blocking checkpointing [10,11,12]. According to the triggering occasion of checkpoints, they can be divided to checkpointing based on time [3] and checkpointing induced by communication [15,16,17,18].

Asynchronous checkpointing, or independent checkpointing, is the method where every process takes its own checkpoint independently. This could avoid sending the coordination messages for checkpointing, but it has the disadvantages such as cascade rollback, higher storage space and longer recovery time.

Mobile computing environments are typical wireless network applications. They have features such as mobility, limited resources and high failure probability, so the traditional distributed checkpointing algorithms can’t be applied to this environment very well. Neves et al. [19] put forward an adaptive algorithm which considers the quality of a wireless network. This algorithm proposed the concept of soft checkpoints and hard checkpoints. Soft checkpoints are stored in the MH_s_, while hard checkpoints are stored in the stable storage of fixed network hosts (such as MSSs). After taking the number of maxSoft soft checkpoints every time, the process takes one hard checkpoint. The advantage of this method is that the value of maxSoft changes adaptively with the server quality of the network, but the MH_s_ will store too many soft checkpoints and thus occupy a lot of storage space when the server quality of network in this method is worse. Some papers [20,21,22] adopted mobile agents to partake in the tasks of MSSs, but the existence of agents will cause increments in the message retransmissions and communication delays. Taking the mobility of wireless terminals into consideration, George et al. [18,23] proposed a checkpointing algorithm based on movement and logs. Namely, MH_s_ take checkpoints independently when they pass a certain number of handoffs. However, this method has longer recovery time when the handoff rate of MH_s_ is low. Cao et al. [18,24] came up with the mutable checkpoint which is stored in MH_s_. The shortcoming of this strategy is that the number of mutable checkpoints induced by messages is large and most of these mutable checkpoints may become useless checkpoints.

Recently, the related researches for ad hoc network are mainly focused on aspects such as the technologies of channel switching, network architecture, routing protocols, strategies for assuring Quality of Service (QoS), multicast protocols, energy conservation and security [22,25,26,27,28,29,30,31], but studies about the fault tolerance and reliability of ad hoc networks are relatively few. Tuli et al. [32] proposed an asynchronous checkpointing strategy which was applied to an ad hoc network, namely MH_s_ could take checkpoints independently. In order to reduce the occupation of storage space in MH_s_, the cluster heads store the message logs for MH_s_. The advantage of this strategy is that MH_s_ could take checkpoints independently and so the communication overhead of the system without failure is small, but the storage overhead of cluster heads is large. When some process fails, the wireless communication overhead to determine the dependence relation of rollback will also be large. Meng et al. [33] put forward a synchronous algorithm for ad hoc networks based on clusters. The basic notion of this algorithm is that the initialization of the checkpointing procedure and the sending of checkpoint requests to MH_s_ in clusters are charged by the cluster heads. If MH_s_ achieve the channels assignment in the first beacon interval, they will take checkpoints in the second beacon interval. Otherwise, they take checkpoints in the first beacon interval. Though this algorithm makes very good use of the channels assignment mechanism of mobile ad hoc networks, the taking of checkpoints is too frequent and the storage overhead is large. Parveen et al. [7] also presented a synchronous checkpointing strategy for ad hoc networks. This strategy assumes that the executions of processes are deterministic, namely the same results will be obtained when the same message sequences are executed twice from the same starting point. Saluja and Kumar [34] proposed a synchronous non-blocking checkpointing strategy, which was suitable for ad hoc networks. In this strategy, the cluster heads substitute for processes in the same cluster to execute the checkpointing duties. When the cluster heads receive the messages, which are sent to the processes in the same cluster, they firstly obtain the dependence relation carried by messages and then send the messages to processes alone.

Though the above checkpointing algorithms for mobile ad hoc networks take the clustering structure of the ad hoc network into account, the burden of cluster heads (cluster heads are MH_s_ too in fact) is too heavy and they consider less the effects caused by the features of the ad hoc network such as multi-hop routing and dynamic topological structure, hence they cannot be applied to ad hoc networks very well.

### 1.3. Work and Organization of This Paper

This paper intends to design a hybrid checkpointing strategy based on a multi-frequency hierarchy ad hoc network. In this strategy, the MH_s_ in the same cluster use the synchronous checkpointing strategy, while the MH_s_ in different clusters make use of the asynchronous checkpointing strategy. The main contributions of this paper include the following:

1. It proposes the hybrid checkpointing model combined the coordinated and uncoordinated checkpointing after considering the communication features and kinds of resource restraints of mobile ad hoc networks;
2. It discusses the checkpoint correctness criteria for a hybrid checkpointing model including the checkpoint taking criteria, rollback criteria and indelibility criteria.
3. It discusses the triggering occasions of checkpoints for processes in the same cluster and different clusters according to the characteristics and requirements of different types of checkpoints.
4. It designs the intra-cluster and inter-cluster checkpoint dependence graph according to the dependence relations among checkpoints. Then it discusses the elimination rules of checkpoints in the same cluster and different clusters based on these graphs. What is more, it provides the algorithms for checkpoints in the same cluster and different clusters and rollback recovery algorithm. At the same time, it proves the correctness of the algorithms by theory.
5. By testing the performances of the proposed strategy, this paper verifies that the hybrid checkpointing strategy is a good trade off method and it has advantages such as less of dependence on cluster heads, low overhead and shorter recovery times.

The rest of the paper is organized as follows: Section 2 introduces the ad hoc network topological structure and the model of hybrid checkpointing. The correctness criteria for hybrid checkpointing model and the triggering occasions of kinds of checkpoints are discussed in Section 3 and Section 4. In Section 5 and Section 6, we propose the elimination strategy of checkpoints and related algorithms. Then we discuss the processing method for handoffs in Section 7. In Section 8, we present the testing of performance and analysis. The last part gives the conclusions of this paper and proposes future work.

## 2. Ad Hoc Network Structure and Hybrid Checkpointing Model

### 2.1. Mobile Ad Hoc Network Topological Structure

The mobile ad hoc network is a kind of wireless self-organizing network which has peer to peer movement. Its topological structures are mainly classified to two kinds: flat structures and hierarchical structures [35,36,37,38]. In a flat structure, all network nodes are equal, but the entire network in the hierarchical structure is constituted by subnets which are divided into clusters. Each cluster includes one cluster head and many cluster members. The cluster heads form a higher-level network. Because the scale of the hierarchical structure is not limited and the expandability of this structure is good, this structure has become the favorite development trend of mobile ad hoc networks, therefore, this paper will discuss the checkpointing algorithm under the multi-frequency hierarchical structure.

**Figure 1:**
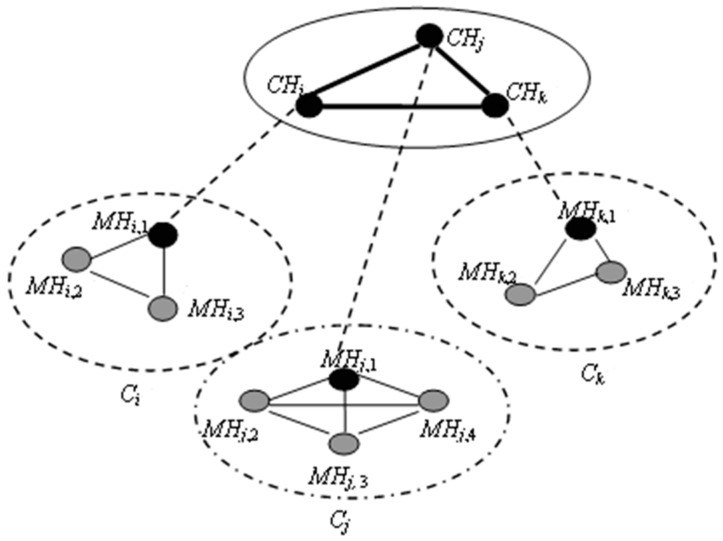
Multi-frequency hierarchy topological structure.

Figure 1 is the multi-frequency hierarchical topological structure of an ad hoc network. This structure consists of multiple clusters. Each cluster includes one cluster head (CH) and a limited number of cluster members [39,40,41]. The CH_s_ and cluster members are all regular MH_s_. The hosts in one cluster share one frequency to communicate, while the CH_s_ communicate on another frequency. The members in she ame cluster can communicate with each other directly, while the members in different clusters have to communicate by the retransmission of CH_s_.

### 2.2. Hybrid Checkpointing Model

As we know, the different kinds of resources on MH_s_ are quite limited in mobile ad hoc networks. These resources include the storage space, processing capacity, battery energy, wireless bandwidth and so on. If we fully adopt the asynchronous checkpointing algorithm, it will need to transmit a lot of checkpoint coordination messages. Because the communication among MH_s_ in different clusters usually needs multi-hop routing before reaching the destination, the number of checkpoint coordination messages is proportional to the number of hops and the retransmission will occupy precious wireless bandwidth. Though it will avoid a lot of checkpoint coordination messages if we completely use the asynchronous checkpointing algorithm, the cascade rollback caused by asynceshronous checkpointing would waste resources such as precious CPU allocation and power sourcs of MH_s_ and the storage space for storing checkpoint information is large.

Therefore, this paper plans to adopt the hybrid checkpointing model which combines the synchronization and asynchronization after considering the kinds of resource limitations in MH_s_ synthetically. In this model, the hosts in the same cluster use the synchronous checkpointing (because these hosts could communicate with each other directly without the retransmissions involving cluster heads), while the hosts in different clusters adopt the asynchronous checkpointing. This will not only avoid the waste of all kinds of resources for hosts in same cluster because of cascade rollback, but also avoid too many transmissions of messages among hosts in different clusters. What is more, it reduces the communication delay of the network and checkpoint operation delay.

**Definition** **1.** A same cluster checkpoint (SC) is a checkpoint which is used for ensuring the consistency of processes in hosts of the same cluster.

The SC is a synchronous checkpoint. Every time the process use a SC, it needs to remain synchronous with the related processes in the same cluster. During the synchronization procedure, if part of processes cannot finish their checkpoints because of their own reasons, then the coordinated checkpoints become useless checkpoints which need to be eliminated. Hence, in order to reduce the occupation of precious storage space in wireless terminals, the SC_s_ can be further divided into two kinds: uncertain checkpoints and certain checkpoints.

**Definition** **2.** An uncertain checkpoint is a SC which is stored in the cache of a MH when the coordination is not finished.

**Definition** **3.** A certain checkpoint is a SC which is stored on the local disk of a MH when the coordination is finished.

Therefore, if and only if the coordination of checkpoints succeeds, the uncertain checkpoints could be turned to certain checkpoints. Otherwise, the uncertain checkpoints become useless checkpoints which could be eliminated. The elimination of uncertain checkpoints involves releasing the information stored in the cache.

**Definition** **4.** A different cluster checkpoint (DC) is a checkpoint which is used for assuring the consistency of processes in MHs of different clusters.

The DC is an asynchronous checkpoint. When a process receives messages from MH_s_ of different clusters, it finishes the checkpoint operation independently without informing other MH_s_. The DC is triggered by the reception of messages from MH_s_ of different clusters and its checkpoint information is directly stored in the local disk of the MH, so the DC is a certain checkpoint in nature.

**Figure 2:**
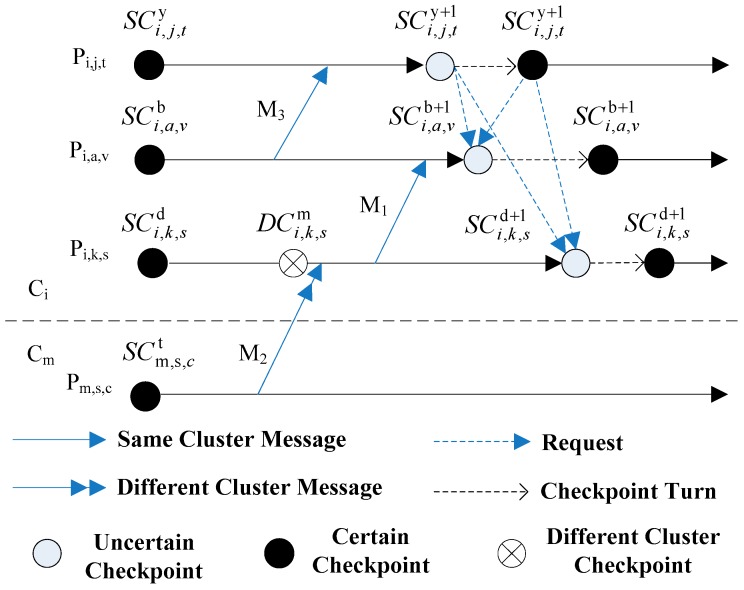
The model of hybrid checkpointing.

Suppose *P_i,j,k_* expresses a process, *i* is the id of cluster which the mobile terminal stays, *j* is the id of mobile terminal and *k* expresses the id of process. Figure 2 is an example of hybrid checkpoint model. *P_i,k,s_*, *P_i,a,v_* and *P_i,j,t_* are the processes executing in different terminals of cluster *C_i_* respectively. While *P_m,s,c_* is the process executing in terminal s of cluster *C_m_*. ${SC}_{i,a,v}^{b}$ expresses the *SC* of *P_i,a,v_* and *b* is the sequence number of checkpoint. ${DC}_{i,k,s}^{m}$ is the *DC* of *P_i,k,s_* and *m* is the id of cluster which the sender of message from different cluster stays.

Combined with the Figure 2, the explanation for principles of hybrid checkpointing model can be expressed as follows:

1. The initialized checkpoint. When each process initializes, it takes an initialization checkpoint (certain checkpoint). For example, ${SC}_{i,a,v}^{b}$, ${SC}_{i,j,t}^{y}$, ${SC}_{i,k,s}^{d}$ and ${SC}_{m,s,c}^{d}$ in Figure 2 are initialized checkpoints.
2. The taking of SC_s_. When a process *P* receives a fixed number of messages from MH_s_ in same cluster, it initializes a coordination procedure of checkpoints. *P* firstly takes an uncertain checkpoint, and then it sends checkpoint requests to the processes which have dependence relation with *P*. When these processes receive the requests, they firstly take uncertain checkpoints and then reply to *P*. By the time *P* receives all replies from processes which take part in the coordination procedure, it turns the uncertain checkpoint into a certain checkpoint. Otherwise, *P* eliminates its own uncertain checkpoint. Finally, *P* notices related processes to finish the turning or elimination operations of checkpoints together.
3. The taking of DC_s_. When a process receives a message from a MH in a different cluster, the DC is triggered. In order to assure that the receiving event of this message can be cancelled when the sending event of this message is cancelled, the process has to take the DC firstly and then receives the message.

In Figure 2, *P_i,j,t_* is the process which initializes the checkpointing. *P_i,a,v_* and *P_i,k,s_* are the participant processes of checkpointing because of messages *M*_2_ and *M*_3_. When the checkpointing starts, *P_i,j,t_* firstly takes a uncertain checkpoint ${SC}_{i,j,t}^{y+1}$ and then notices *P_i,a,v_* and *P_i,k,s_* to take uncertain checkpoints ${SC}_{i,a,v}^{b+1}$ and ${SC}_{i,k,s}^{d+1}$ respectively. By the time *P_i,a,v_* and *P_i,k,s_* finish each uncertain checkpoint ${SC}_{i,a,v}^{b+1}$ and ${SC}_{i,k,s}^{d+1}$, they send replies to *P_i,j,t_*. After *P_i,j,t_* has received the replies from *P_i,a,v_* and *P_i,k,s_* (not marked in Figure 2), *P_i,j,t_* turns ${SC}_{i,j,t}^{y+1}$ to certain checkpoint. In addition, *P_i,j,t_* notices *P_i,a,v_* and *P_i,k,s_* to turn ${SC}_{i,a,v}^{b+1}$ and ${SC}_{i,k,s}^{d+1}$ into certain checkpoints, but their Checkpoint Sequence Number (CSN) does not change. Otherwise, *P_i,j,t_* eliminates its own uncertain checkpoint and notices *P_i,a,v_* and *P_i,k,s_*. For example, when process *P_i,k,s_* in cluster *C_i_* receives a message *M*_2_ from process *P_m,s,c_* in cluster *C_m_* as Figure 2 shows, *P_i,k,s_* firstly takes ${DC}_{i,k,s}^{m}$ and then receives *M*_2_.

### 2.3. Problem of Hybrid Checkpointing Model to Be Solved

The hybrid checkpoint model takes both of the communication features of hosts in same cluster and different clusters into consideration, and thus reduces the number of wireless communication messages greatly and saves the kinds of resources of MH_s_. What is more, this model has less dependence on cluster heads (it just records the dependence among checkpoints of processes in different clusters, and it will be discussed in Part 5). Therefore, this model has better flexibility.

However, in a hybrid checkpointing strategy, the checkpoints of a process cannot be handled according to the fully synchronous or asynchronous checkpointing algorithm because of the existing of *SC_s_* and *DC_s_*. The main problems for checkpoints processing are as follows:

1. What are the correctness criteria? In the hybrid checkpoint model, because there are mutual dependence relations among checkpoints in the same cluster and different clusters, so it is necessary to research a brand new global consistency criterion for checkpoints to assure the global consistency of checkpoints.
2. When is the checkpoint triggered? In order to reduce the checkpoint operations and storage overhead, different triggering occasions should be taken for different kinds of checkpoints according to the ad hoc network features and hybrid checkpointing model.
3. When is the checkpoint eliminated? The existing of *DC_s_* makes the elimination of *SC_s_* be complicated, and so creates a lot of checkpoints. In order to save space, it needs to clear away the useless *SC_s_* in time and coordinate the useless *DC_s_* actively.
4. What is the strategy for processing handoff? The checkpointing strategy for the hybrid checkpoint model is decided by the same cluster and different clusters relations of MH_s_. However, the same cluster and different clusters relations of MH_s_ will change when MH_s_ take handoff. Hence, the checkpointing strategy should dispose the change of MH_s_ correctly and automatically maintain the new cluster relation to guarantee the correct checkpoint operations of processes in handoff MH_s_.

## 3. Correctness Criteria for a Hybrid Checkpointing Model

In the asynchronous checkpointing strategy, the condition that checkpoints ensure the global correctness is no orphan messages. An orphan message is a message whose receiving event is stored but its sending event is not stored. Therefore, in order to assure the messages not become orphan messages, the sender process of the message must take a checkpoint also when the receiver process of a message takes a checkpoint, but in the asynchronous checkpointing strategy, the orphan messages are allowed to exist. Hence, the cascade rollback phenomenon exists. However, the checkpoints in the same cluster and different clusters adopt different strategies. Therefore, it could not finish the checkpoint operation according to the checkpoints correctness criteria of coordinated or uncoordinated strategy purely.

Let the same cluster message (SM) be the message transmitted from process *SP_i_* to process *SP_j_* of MH in same cluster. *SC_i_* and *SC_j_* are respectively the checkpoints taken by *SP_i_* and *SP_j_* after sending and receiving SM. The different cluster message (DM) is the message transmitted between from process *DP_i_* to process *DP_j_* of MH in another cluster. *CKP_i_* and *DC_j_* are respectively the checkpoints of *DP_i_* and *DP_j_* related to DM. The Receive(*M*) is the operation of receiving message *M* and the Send (*M*) expresses the operation of sending message *M*. *A* >> *B* expresses that the appearance of operation *A* is prior to operation *B*. *CKP* means one checkpoint (SC or DC).

### 3.1. Checkpoint Taking Criteria

For assuring the SM is not an orphan message, it requires that the sending process of SM must take a checkpoint when the receiving process of SM takes a checkpoint:

**Criterion** **1:** ∀ *SM*, if Receive(*SM*) >> *SC_i_* meets, then Send(*SM*) >> *SC_j_* must meet it also.

The DC does not require assuring no orphan message, but it requires that the receiving event of DM could be cancelled when the sending event of DM is cancelled. Hence, it demands that the receiving process of DM firstly takes a DC and then receives the message.

**Criterion** **2:** ∀ *DM*, if *CKP_i_* >> Send(*DM*) meets, then *DC_j_* >> Receive(*DM*) must meet it also.

### 3.2. Checkpoint Rollback Criteria

To guarantee that all processes could roll back to the global consistent checkpoint states when some processes fail, it requires that the receiving event of message could be cancelled when the sending event of message is cancelled. This requirement is suitable to both SC_s_ and DC_s_:

**Criterion** **3:** ∀*SM*, if ∃*SC_j_*−1(*SC_j_*−1 >> Receive(*SM*)>>*SC_j_*) ∧ ∃*SC_i_*−1(*SC_i_*−1 >> Send(*SM*) >> *SC_i_*), then *SP_j_* must be able to roll back to *SC_j_*−1 when *SP_i_* rolls back to *SC_i_*−1.

**Criterion** **4:** ∀*DM*, if *DP_i_* rolls back to *CKP_i_*, then *DP_j_* must be able to roll back to *DC_j_*.

### 3.3. Checkpoint Indelibility Criteria

In synchronous checkpointing strategy, the processes which joins the checkpointing can clear away the prior checkpoint when the checkpointing is finished. While the processes in asynchronous checkpointing strategy will eliminate checkpoints together after finding the global consistent checkpoint and finishing rollback recovery when some process fails, however, the checkpoints of one process not only include the SC_s_ related to MHs in same cluster, but also contain the DC_s_ related to MHs in different clusters in a hybrid checkpoint environment. When a process finishes the SC, the other SC_s_ earlier than this SC cannot be eliminated easily, otherwise, global inconsistency may appear. The elimination of checkpoints is judged by the relations among checkpoints:

**Definition** **5.** *If CKP_i_ >> Send(M) and CKP_j_ >> Receive(M), then CKP_j_ directly depends on CKP_i_ between processes. It expresses as CKP_j_* ⇒ *CKP_i_*.

**Definition** **6.** *Let CKP_i_ and CKP_i+1_ to be the contiguous checkpoints of P_i_, and then CKP_i+1_ directly depends on CKP_i_ in same process. It expresses as CKP_i+1_* ⇒ *CKP_i_*.

The upper two dependence relations are all named by checkpoint direct dependence relation. They will not be distinguished in the later parts:

**Definition** **7.** *If CKP_i_* ⇒ *CKP_j_ and CKP_j_* ⇒ *CKP_k_, then CKP_i_ depends on CKP_k_ indirectly. It expresses as CKP_i_* ⇒ ⇒ *CKP_k_.*

**Figure 3:**
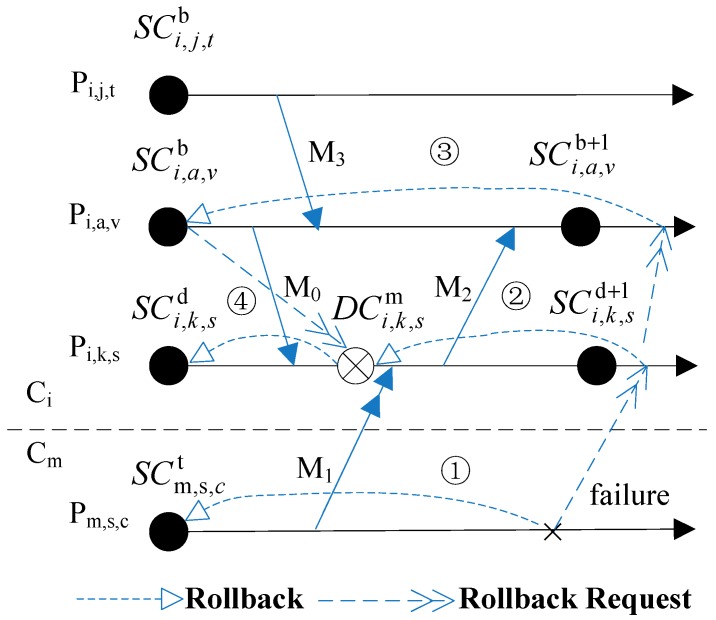
Example of checkpoints dependence.

In Figure 3, there are relations such as ${SC}_{i,a,v}^{b}$ ⇒ ${DC}_{i,k,s}^{m}$ and ${SC}_{i,k,s}^{d}$ ⇒⇒ ${DC}_{i,k,s}^{m}$. If *P_i,a,v_* and *P_i,k,s_* clear away ${SC}_{i,a,v}^{b}$ or ${SC}_{i,k,s}^{d}$ at once after finished ${SC}_{i,a,v}^{b+1}$ or ${SC}_{i,k,s}^{d+1}$, then *P_i,a,v_* or *P_i,k,s_* will not be able to roll back to global consistent states when *P_m,s,c_* rolls back to ${SC}_{m,s,c}^{t}$ (The correct rollback sequence is ①→②→③→④).

Let *SC_i_* and *SC_k_* be the SC_s_ of process *P*. *DC_x_* is the DC of *P* or the processes in the cluster same as *P*. Then the indelibility criteria for checkpoint can be described as follows:

**Criterion** **5:** If ∃*DC_x_* (*SC_j_* ⇒ *DC_x_* or *SC_j_* ⇒ ⇒ *DC_x_*) ∧ ∃*SC_k_* (*k*>*i*), then *SC_j_* cannot be eliminated.

**Figure 4:**
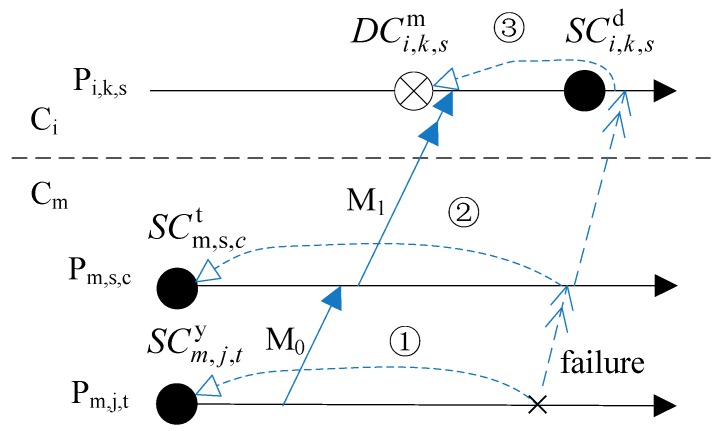
Example of rollback in different clusters.

Criterion 5 indicates that if one SC which is not newest depends on any DC directly or indirectly then it cannot be eliminated. Because the DC is an asynchronous checkpoint, it may cause a cascading rollback. As Figure 4 shows, the rollback of processes *P_m,s,c_* and *P_m,j,t_* will all cause *P_i,k,s_* rolling back to ${DC}_{i,k,s}^{m}$ (rollback ① and ② all cause the rollback ③). If ${DC}_{i,k,s}^{m}$ is eliminated earlier than ${SC}_{m,s,c}^{t}$ and ${SC}_{m,j,t}^{y}$, then *P_i,k,s_* will not be able to roll back to the consistent states with processes in *C_m_* when *P_m,s,c_* rolls back to ${SC}_{m,s,c}^{t}$ or *P_m,j,t_* rolls back to ${SC}_{m,j,t}^{y}$.

Therefore, the indelibility criteria of DCa can be defined as follows:

**Criterion** **6:** If *CKP_i_* is not eliminated, then *DC_j_* must not be cleaned up.

Criterion 6 shows that if the checkpoints of MH_s_ in different clusters which are dependent on one DC are not cleaned up, then this DC cannot be eliminated also. Otherwise, it will cause something wrong about rollback.

**Criterion** **7:** If *CKP_i_* has been eliminated, then *DC_j_* cannot be cleared away when one of the following conditions is satisfied.

*DC_j_* is the only checkpoint of *DP_j_*. *DC_j_* does not depend on the checkpoints of *DP_j_*, but it depends on the checkpoints of processes in the same cluster of *DP_j_*.

Th Criterion 7 means that if one DC is the only checkpoint of process or the DC is the earliest checkpoint and the SC_s_ on which the DC depends are not eliminated, then the DC cannot be cleared away. The former requires that the new state of process be stored in an update checkpoint, while the later demands that the elimination of a DC will not cause wrong rollback. Hence, there will be one conclusion: if the hybrid checkpointing strategy satisfies the Criteria 1~7, then this strategy could assure the global consistency of checkpoints.

## 4. Checkpoint Triggering Occasions

In the distributed system, the checkpoint always adopts the way to be triggered at a fixed time or by a fixed number of messages. For the ad hoc network, the time interval for triggering checkpoints periodically is hard to decide because of the dynamism and uncertainty of this network on the one hand, and on the other hand, a timing trigger may usually wake up some MHs staying dormant state and then cause unnecessary battery consumption. Therefore, the SC_s_ and DC_s_ in the hybrid checkpointing model all adopt the active trigger mechanism based on messages. Besides, the SC_s_ use the reactive trigger mechanism to reduce the number of *SC_s_*, which cannot be eliminated. There are two different SC triggers: Active Trigger based on Message and Reactive Trigger:

1. Active Trigger based on Message: The active trigger means that a process initializes a checkpointing procedure actively when it receives *K* (*K* is the threshold, *K* ≥ 1) messages from MH_s_ in same cluster. The value of *K* is directly proportional to the continued communication time without failure and storage space of the MH, but inversely proportion to the disconnection and failure probability of the MH. What is more, the choice of *K* should consider the kinds of overhead of the ad hoc network. This part will be further discussed and verified in the experiments.
2. Reactive Trigger: It can be seen from Criterion 5, that if one SC depends on any DC directly or indirectly then it cannot be cleared away, so there will be a lot of SC_s_ which could not be eliminated.

**Figure 5:**
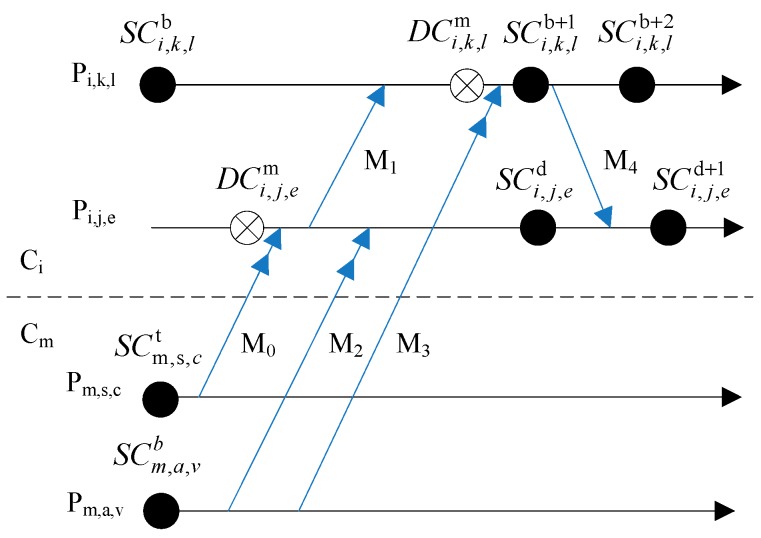
An example of the coexistence of multiple same cluster checkpoints.

As Figure 5 shows, all SC_s_ of *P_i,k,l_* and *P_i,j,e_* depends on ${DC}_{i,j,\varepsilon }^{m}$ directly or indirectly, so they could not be cleared away before ${DC}_{i,j,\varepsilon }^{m}$ is eliminated. If the checkpoint frequency of cluster *C_i_* is higher than the one of *C_m_*, then there may be a lot of SC_s_ of *C_i_* which cannot be cleaned up.

The key to solve this problem is to clean up the DC_s_ on which the SC_s_ depend. According to the Criteria 6 and 7, the premises to eliminate DC include two conditions. One is that the process has finished new checkpoint (namely this DC is not the new checkpoint of the process). The other is that the checkpoints of processes in other clusters on which the process depends have been eliminated.

Let *C_i_* and *C_j_* to be two different clusters. If the processes in *C_i_* which contains all DC_s_ (created by the receiving of messages from MH_s_ in *C_j_*) related to *C_j_* have finished the certain checkpoints (namely meet the first premise), then *C_i_* notices *C_j_* to initialize a new checkpointing (only the new checkpoint is done, the former SC_s_ can be cleaned up and this meets the last premise). If some checkpoint dependent on the DC_s_ in *C_i_* can be cleared away after the related processes in *C_j_* has finished new *SC*, then the elimination probability of the DC_s_ increases heavily. Then the SC_s_ in *C_i_* which depend on the DC_s_ could be cleaned up further. The detailed elimination rules of checkpoints will be discussed in Section 5.

Different from the active trigger, this checkpointing procedure is initialized by the CH and the related processes in cluster just act as the participants, so it is called a reactive trigger.

The DC uses the message trigger mechanism also, namely the DC is triggered when it receives messages from MH_s_ in different clusters. However, if a process takes a DC when it receives a message from the same different cluster each time, it will increase the checkpoints and so augment the storage overhead heavily.

**Figure 6:**
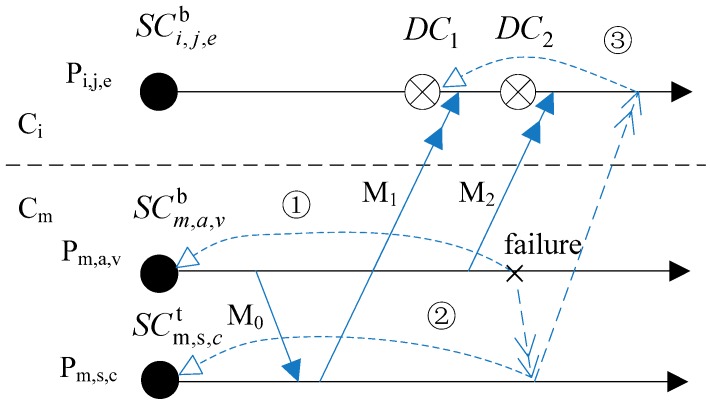
Example of DC taking.

As Figure 6 shows, *P_i,j,e_* in cluster *C_i_* receives messages *M*_1_ and *M*_2_ from processes *P_m,s,c_* and *P_m,a,v_* in cluster *C_m_* respectively. If *P_i,j,e_* takes *DC*_1_ and *DC*_2_ respectively, then *P_i,j,e_* will roll back according to the following situations when processes in *C_m_* fail.

*M*_0_ exists. If *P_m,a,v_* rolls back to $C_{m,s,c}^{b}$ because of failure, *P_m,s,c_* will roll back to ${SC}_{m,s,c}^{b}$ and *P_i,j,e_* should roll back to *DC*_1_; if *P_m,s,c_* rolls back to ${SC}_{m,s,c}^{b}$ because of failure, *P_i,j,e_* will roll back to *DC*_1_ also.

*M*_0_ does not exist. If *P_m,a,v_* rolls back to $C_{m,a,v}^{b}$ because of failure, *P_i,j,e_* will roll back to *DC*_2_; if *P_m,s,c_* rolls back to ${SC}_{m,s,c}^{b}$ because of failure, *P_i,j,e_* will roll back to *DC*_1_.

It can be seen, *P_i,j,e_* will roll back to *DC*_1_ in most instances. Hence, for reducing the number of DC_s_, a process just takes one DC for each different cluster during one DC interval.

## 5. Strategies for Checkpoint Elimination

**Figure 7:**
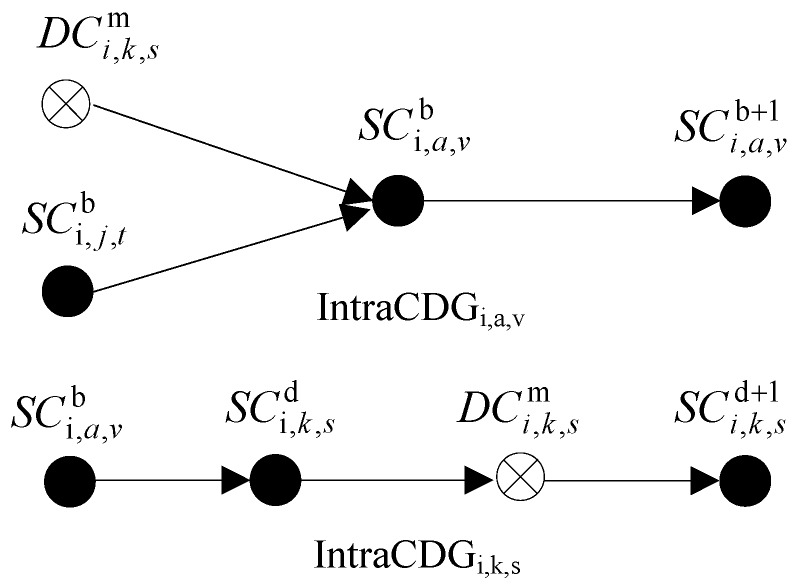
IntraCDG_i,a,v_ and IntraCDG_i,k,s_.

The storage space of MH_s_ in an ad hoc network is quite limited, so it is necessary to clean up the overdue useless checkpoints in time. This section will firstly introduce the checkpoint dependence graph, and then design the relevant rules and strategies for checkpoint elimination. The Figure 7 below shows the *IntraCDG* of processes *P_i,a,v_* and *P_i,k,s_* (see in Figure 3). Because there are no dependence relations in proesses *P_m,s,c_* and *P_i,j do,t_*, they not have the dependence relations at present and so the *IntraCDG_m,s,c_* and *IntraCDG_i,j,t_* are empty.

### 5.1. Checkpoint Dependence Graph

#### 5.1.1. Intra-Cluster Checkpoint Dependence Graph

In order to describe the dependence relations among checkpoints of processes in same cluster clearly, every process stores an Intra-cluster Checkpoint Dependence Graph (*IntraCDG*). The nodes of *IntraCDG* are certain checkpoints or DC_s_ and the edges of *SDG* are directed edges. The edge is started by one checkpoint and ended by another checkpoint on which the checkpoint depends. According to Definitions 5 and 6, the update situations of *IntraCDG* contain the following two aspects:

1. The update of dependence relations among processes. When a process *P_i_* receives a SM, it firstly obtains the recent checkpoint of the sender process and then checks whether the edge started by this recent checkpoint and ended by current checkpoint of *P_i_* exists in *IntraCDG_i_*. If the edge does not exist, then it will be added to *IntraCDG_i_*.
2. The update of dependence relations in same process. When *P_i_* takes a new certain checkpoint or DC, it adds a directed edge started by the current checkpoint (a certain checkpoint or DC) and ended by the new checkpoint to *IntraCDG*.

#### 5.1.2. Inter-Cluster Checkpoint Dependence Graph

In order to describe the dependence relations among checkpoints of processes in different clusters, the source cluster head and target cluster head store Inter-Cluster Checkpoint Dependence Graph (InterCDG) for sending messages and receiving messages. They are marked by *S_InterCDG* and *R_InterCDG,* respectively. The nodes of *InterCDG* are certain checkpoints or DC_s_ and the edges of it are directed edges. The edge is started by one checkpoint and ended by another checkpoint on which the checkpoint depends.

In Figure 4, ${DC}_{i,k,s}^{m}$ depends on ${SC}_{m,s,c}^{t}$. This dependence relation can be recorded in the *InterCDG_s_* related to *M*_1_. The *InterCDG_s_* are respectively stored in *CH_i_* and *CH_m_*. The figure is comparative simple, and so it is not given.

### 5.2. Rules and Strategies of Checkpiont Elimination

The checkpoint elimination includes two parts: the elimination of SC and elimination of DC.

#### 5.2.1. Elimination of *SC*

As Criterion 5 shows, one SC can be cleared away if it is not the newest checkpoint of process and it does not depend on any DC.

Let *IntraCDGglobal* to be the global checkpoint dependence graph of all processes in one cluster. *SC_i_* and *SC_k_* are the *SC_s_* of *P_i_*. Set-Path(*SC_i_*) is the set of paths which are ended by *SC_i_* in *IntraCDGglobal*. *DC_x_* is any DC. The elimination rule *of SC can* be described in formalized way as follows:

Let *P_i_* to be the process which cleans up the *DC_x_*, then the algorithm for elimination of DC is indicated as Algorithm 1.

**Algorithm 1 DC_Elimination( )****if** (∃Edge(*CKP_i_*, *DC_x_*) ∈ Intra*CDG_i_*) **then** DELETE(*DC_x_*); REPLACE_IntraCDGnodes(*CKP_i_*, *DC_x_*); // *CKP_i_* replaces *DC_x_* NOTICE_IntraCDGchange(*CKP_i_*, *DC_x_*); //Notice processes the replacement of *CKP_i_* for *DC_x_* **else** **if** (∃Edge(*DC_x_*, *CKP_i_*) ∈ *IntraCDG_i_*) **then** **if** (∃Edge(*CKP_j_*, *DC_x_*) ∈ Intra*CDG_i_* ∧ *CKP_j_* ∉ *P_i_*) **then** TurnDCtoSC(*DC_x_*, *SC_k_*); // Turn *DC_x_* to *SC_k_* NOTICE_CKPturn(*DC_x_*, *SC_k_*); //Notice processes in same cluster of turning of *DC_x_* **else** DELETE(*DC_x_*); NOTICE_delete(*DC_x_*); //Notice processes in same cluster of the elimination of *DC_x_* //Notice processes in same cluster of the elimination of *DC_x_*

**Rule** **1:** If ∄*DC_x_* ∈ Set-Path(*SC_i_*) ∧ ∃*SC_k_* (*k* > *i*), then *SC_i_* can be cleaned up.

The elimination occasions of *SC* include two occasions:

1. When one process finishes the new SC, the initialization process of checkpointing (active trigger) or the CH (reactive trigger) will collect the *IntraCDGglobal* and check whether the earlier checkpoints can be cleaned up.
2. After any DC is cleared away by one process, the process will notice the CH to collect the *IntraCDGglobal*. Then the CH checks whether the earlier checkpoints can be cleaned up.

#### 5.2.2. Elimination of *DC*

It can be seen from Criterion 6, one *DC* must not be able to be cleared up if the related checkpoints of processes in other clusters on which the DC depends directly are not eliminated. Hence, one premise of DC to be cleaned up is that all checkpoints from other clusters on which the DC depends directly are cleared away. However, it needs to check whether the elimination of DC satisfies the conditions in Criterion 7 even if this premise meets. The elimination of DC_s_ is based on the elimination of SC_s_. If the conditions are satisfied, then the DC still cannot be cleared away.

Let *DC_x_* to be any DC of process *P_i_*. *CKP_i_* is any kind of checkpoint of *P_i_*. *CKP_j_* is any checkpoint of process in the cluster same as *P_i_*. Edge(*A*, *B*) expresses the edge started by *A* and ended by *B* in *IntraCDG*. Then the elimination rules of *DC* can be described in formalized way as follows:

**Rule** **2:** If ∃Edge (*CKP_i_*, *DC_x_*) ∈ *IntraCDG_i_*, then *DC_x_* can be cleaned up.

**Rule** **3:** If ∃Edge (*CKP_i_*, *DC_x_*) ∉ *IntraCDG_i_*, then if only if ∃Edge(*DC_x_*, *CKP_i_*) ∈ *IntraCDG_i_* ∧ ∃Edge (*CKP_j_*, *DC_x_*) ∉ *IntraCDG_i_*), then *DC_x_* can be cleaned up.

Because the CH to which the sending process of DM belongs records *S_IntraCDG*, so the process needs to inform the CH to delete the checkpoint and related edges in *S_IntraCDG* when the process cleans up the SC. If the CH finds the isolated DC *DC_x_*, then it informs the related cluster to judge whether the DC can be eliminated according to Rules 2 and 3. After receiving the processing request of the DC, the related process decides the next step operation by its own *IntraCDG*.

1. If there is any edge which is started by checkpoint *CKP_i_* of process and ended by *DC_x_* in *IntraCDG*, then *DC_x_* will be cleared away. The edge which is started by and ended by *DC_x_* will be eliminated. What is more, it will use *CKP_i_* to replace *DC_x_*. Finally, the CH informs the processes in same cluster to change the *DC_x_* to *CKP_i_* in their own *IntraCDG_s_*.
2. If there is no edge started by the own checkpoint *CKP_i_* of process and ended by *DC_x_*, and there is edge started by *DC_x_* and ended by its own checkpoint *CKP_j_*, then two situations exist.
3. If there is not edge started by checkpoint *CKP_a_* of process in same cluster and ended by *DC_x_* in *IntraCDG*, then can be cleared away.
4. If there is edge started by checkpoint *CKP_a_* of process in same cluster and ended by *DC_x_* in *IntraCDG*, then turns *DC_x_* to *SC_k_* and informs other processes in same cluster to change *DC_x_* in *IntraCDG* to *SC_k_*.

**Theorem** **1.** The strategy of checkpoint elimination satisfies the correctness criteria of hybrid checkpointing model.

**Proof.**  We prove this from the SC and DC, respectively:

1. Let *SC_i_* and *SC_j_* to be the SC_s_ of any process *P_i_*. *DC_x_* is any DC. According to Rule 1, if there is no path from *DC_x_* to *SC_i_* but there is node *SC_j_* (*j* > *i*) in *IntraCDGglobal*, then it shows that *SC_i_* is not the current checkpoint of *P_i_* and there is no *DC_x_* meeting the condition *SC_i_* ⇒ *DC_x_* or *SC_i_* ⇒⇒ *DC_x_*. At this moment, any rollback of process in different cluster will not cause *P_i,j,t_* to roll back to ${SC}_{i,j,t}^{y}$. Hence, *SC_i_* can be eliminated. It can be seen, the elimination of *SC_s_* meets the Criterion 5.
2. Let the cluster to which the DC belongs to be *C_m_*. If there is any isolated *DC_x_* in S_*InterCDG_m_* after *CH_m_* has handled the set of checkpoints that could be eliminated, then it means that any checkpoint *CKP_i_* of the process in *C_m_* does not meet the condition *DC_x_⇒ CKP_i_.* Therefore, any rollback of process in *C_m_* will not cause *P_i_* to roll back to *DC_x_*.

Let *CKP_a_* to be any kind of checkpoint of *P_i_*. *CKP_b_* is any kind checkpoint of process in the cluster same as *P_i_*. If ∃Edge(*CKP_a_*, *DC_x_*) ∈ *IntraCDG_i_*, when *DC_x_* has no relation with checkpoints in *C_m_*, then *P_i_* can roll back to *CKP_a_* relatively. Hence, the elimination of *DC_x_* will not cause wrong rollback. If ∃Edge (*CKP_a_*, *DC_x_*) ∈ *IntraCDG_i_* and ∃Edge(*DC_x_*, *CKP_a_*) ∈ *IntraCDG_i_* ∧ ∃Edge (*CKP_b_*, *DC_x_*) ∈ *IntraCDG_i_*, then it shows that no rollback will cause *P_i_* to roll back to *DC_x_* but there is checkpoint which stores the newest state of *P_i_*. Hence, *DC_x_* can be eliminated. It can be seen that the elimination of *DC_s_* meets the Criteria 6 and 7. In conclusion, the strategy of checkpoint elimination satisfies the correctness criteria of the hybrid checkpointing model.

## 6. Checkpointing and Recovery Algorithms

### 6.1. Algorithm for Checkpoints in the Same Cluster

Different from the traditional synchronous checkpointing strategy, when a process initializes the checkpointing, it firstly collects the dependence list of each process in the same cluster to obtain the dependence relation among processes. Then it finds out the processes that have to remain synchronous with it for realizing the checkpoints coordination of processes. However, if there are multiple processes initializing the checkpointing in the same cluster at the same time, then it will create unnecessary wireless communication overhead. Hence, let the CH of each cluster to control one token. In order to avoid the concurrent initializations, only the process which gets the token from CH is allowed to initialize the checkpointing. The all processes staying in active states in one cluster is named as the process list (PL).

In order to reduce the occupation of useless checkpoints to the precious storage space of wireless terminals, this paper divides the SC_s_ into uncertain checkpoints and certain checkpoints (see the Definitions 2 and 3). If and only if the all processes which take part in the checkpointing finish the coordination of checkpoints, the uncertain checkpoints could be turned to certain checkpoints. Otherwise, these uncertain checkpoints will be eliminated, namely the caches released.

Different from the DL which stores the list of processes depended on one process directly, the *LDS* stores the set of processes depended on one process directly and indirectly. The dependence relations in *LDS* can be achieved from *the DL_s_ of* different processes:

**Definition** **8.** *If P_i_ sends a message to P_j_, then P_j_ depends on P_i_ directly. It expresses as P_j_→P_i_*.

**Definition** **9.** *If P_k_→P_j_ and P_j_→P_i_, then P_k_ depends on P_i_ transitively. It expresses as P_k_→P_i_*.

**Definition** **10.** The least dependence set (LDS) of P_i_ is the set of processes in cluster same as P_i_ which depends on P_i_ directly or transitively. If P_j_→P_i_ or P_k_→P_i_, then P_j_, P_k_ ∈ LDS_i_.

To describe the up-dependence relations, the dependence list (DL) is used to store the set of process which directly depends on one process, and so, the transitive dependence relation for *LDS* can be obtained from the *DL_s_* of different processes. The steps of checkpointing in same cluster are described as follows:

1. When process *P* receives *K*, *SM_s_*, it sends token request to CH. If the token is not assigned, then *P* gets the token. Otherwise, *P* goes on applying for the token. During the interval, if *P* receives the checkpoint request from other process, then it takes an uncertain checkpoint.
2. After getting the token, *P* initializes the checkpointing. This procedure includes collecting the DLs of other processes, calculating *LDS* and so on.
3. *P* takes an uncertain checkpoint and sends checkpoint request and *LDS* to all of the other processes in same cluster. If some members stay in the disconnected states, it sends the information to the CH.
4. After getting the checkpoint request, the processes will take uncertain checkpoints if they are part of *LDS*. Then they send replies to *P*.
5. If *P* receives all of the positive replies, then it turns the uncertain checkpoint to certain checkpoint and notices the processes in *LDS* to turn. If some members stay in the disconnected states, *P* sends the turn request to the CH. If *P* does not receive all of the positive replies during a time interval, *P* clears away the uncertain checkpoint and informs all of the processes in the same cluster.
6. If *P* finishes the checkpointing successfully, then *P* collects the *IntraCDG* of processes in same cluster to build the *IntraCDGfinal* and *P* checks whether any checkpoints could be eliminated according to Rule 1. If this kind of checkpoint exists, *P* notices the processes in same cluster to execute elimination processing.

Let *P_j_* to be the process which initializes the checkpointing. *P_j_* stays in the cluster *C_m_*. *PL_m_* expresses the process list of *C_m_*. *CSN_i_* indicates the checkpoint sequence number (CSN) of *P_j_*. *UC_i_* expresses whether *P_j_* has taken the uncertain checkpoint. The *UC_i_* initialization value of is 0. *DP_m_* indicates the set of processes which are disconnected in *C_m_*. *MN_i_* indicates the number of messages received by *P_i_* from processes in same cluster. The algorithm of process which initializes the checkpointing is indicated as Algorithm 2.

**Algorithm 2 CKP_InitProcess( )** Ask_token (*CH_m_*); //ask for token **if** (token is assigned) **then** WAIT (token); **else** *CSN_i_* = *CSN_i_*+1; //increase its own *CSN*
**for** (*P_k_* ∈ *PL_m_* ∧ *k* ≠ *i*) **do** SEND_DLrequest(*P_k_*); //send *DL* request CALCULATE(*LD_S_*, *P_i_*); DO_uncheckpoint(*SC_i_*);  *UC_i_* = 1; //change mark of uncertain *CKP* **for** (*P_k_* ∈ *LDS*) **do** **if** (*P_k_* ∈ *DP_m_*) **then** SEND_CKPReq(*CH_m_*); //send *CKP* request **else** SEND_CKPReq(*P_k_*); **if** (*P_i_* get all replies) **then** CKP_Turning(*SC_i_*); //turn uncertain *CKP* to certain *CKP* **for** (*P_k_* ∈ *LDS*) **do** **if** (*P_k_* ∈ *DP_m_*) **then** SEND_CKPturn(*CH_m_*); //send checkpoint turn request **else** SEND_CKPturn (*P_k_*); FIND_CKPdeleted(*IntraCDGfinal*); //find the deletable checkpoints*/ NOTICE_CKPdeleted();  /*notice processes in same cluster of *CKP* elimination*/ *MN_i_* = 0; /*reset receiving messages number*/

### 6.2. Algorithm for Checkpoints in Different Clusters

The DC is triggered by the message that is sent from a process in a different cluster. The processing of DC is relatively simple. The following steps are its detailed procedures:

1. When a process sends a DM to MHs in different clusters, it adds the current checkpoint to the message.
2. After received the DM, the CH updates *S_InterCDG* according to the dependence relation between checkpoints of the sender and receiver process and then transmits the DM.
3. The CH of the cluster to which the receiver process of the DM belongs updates the *R_InterCDG* also after received the DM, and then transmits the DM to the receiver process.
4. By the time the process received the DM, if it has taken DC for the cluster, it receives the DM directly. Otherwise, it checks whether it receives or sends any message during the current checkpoint interval. If it has sent or received any message, it stores a *OC*. Or else, it turns the current checkpoint to *OC*.

Let DM be a message sent by *P_i_* (belongs to *C_s_*) and received by *P_j_* (belongs to *C_t_*). *CKP_i_* and *DC_j_* are respectively the checkpoints of *P_i_* and *P_j_* related to DM. The algorithm for checkpoints in different clusters is described as Algorithm 3.

**Algorithm 3 DC_Processing( )** SEND_message(*P_i_*, *DM*); // *P_i_* sends *DM* TRANSMIT_message(*CH_s_*, *DM*); // *CH_s_* transmits *DM* UPDATE_S_InterCDG(*P_i_*, *P_j_*); // update *S_InterCDG* TRANSMIT_message(*CH_t_*, *DM*); UPDATE_R_InterCDG(*P_i_*, *P_j_*); RECEIVE_message(*P_j_*, *DM*); // *P_j_* receives *DM* **if** (*P_j_* has taken *DC_j_*) **then** RECEIVE(*DM*); **else** **if** (∄*M*((Send(*M*) >> *CKP_k_*) || (Receive(*M*) >> *CKP_k_*)) **then** TURN_CKP(*CKP_k_*, *DC_j_*); // *CKP_k_* turns to *DC_j_* **else** STORE_CKP(*DC_j_*);

### 6.3. Algorithm for Failure Recovery

When a process fails, it firstly asks related processes to roll back to some global consistency states. Then these processes execute the operations stored in the logs. The keys of the correctness for rollback recovery are that determining the earliest rollback set of process and finding out the processes in different clusters belonging to the cascade rollback.

**Definition** **11.** *The rollback set of P_i_ is the set of checkpoints to which the processes in same cluster and P_i_ may roll back, it is recorded as RS_i_*.

**Definition** **12.** *The earliest rollback set is the set of earliest checkpoints to which the processes in same cluster and P_i_ must roll back, it is recorded as ERS_i_, ERS_i_ ⊆ RS_i_*.

**Definition** **13.** *Let CKP_i_ to be the checkpoint to which the failure process P_i_ needs roll back. If ∃CKP_k_(CKP_k_* ⇒ *CKP_i_* *∨ CKP_k_* ⇒ ⇒ *CKP_i_), then CKP_k_* *∈ RS_i_.*

**Definition** **14.** *Let P_i_ to be the failure process, P_j_ is the process in the cluster same as P_i_. CKP_1_, CKP_2_, …, CKP_n_ are the set of checkpoints of P_j_ and they belong to RS_i_. If ∃CKP_x_, x ∈ {1, 2, …, n}, the condition CKP_x_* ⇒ *CKP_k_ (k ∈ {1, 2*, *…*, *n}, x* ≠ *k) is not satisfied, then CKP_x_ ∈ ERS_i_.*

The ERS calculation procedure for failure process *P_i_* is described as follows:

1. *P_i_* collects the *IntraCDG_s_* of all processes in same cluster and builds the *IntraCDGglobal*.
2. *P_i_* executes the depth-first traversal to the *IntraCDGglobal* started from the checkpoint to which *P_i_* has to roll back, and adds any checkpoints that can be arrived to the *RS_i_*. Finally, the earliest checkpoint of each process in *RS_i_* is added to *ERS_i_*.

Based on the *ERS*, we define the following rollback rules:

**Rule** **4:** Let *P_i_* to be the failure process. *CKP_x_* is any checkpoint of process *P_j_* in the cluster same as *P_i_*. If *CKP_x_* ∈ *ERS_i_*, then *P_j_* should roll back to *CKP_x_*.

**Rule** **5:** Let *P_i_* to be the failure process. *CKP_x_* is any checkpoint of process *P_j_* in the cluster same as *P_i_*. *CKP_x_* ∈ *RS_i_*. If ∃Edge(*CKP_x_*, *DC_x_*) ∈ *S_InterCDG_m_*, then the related process in other cluster will be noticed to roll back to *DC_x_*.

Let *P_i_* to be the failure process and *CKP_i_* to be the checkpoint to which *P_i_* has to roll back. The rollback recovery algorithm is described as Algorithm 4.

**Theorem** **2.** The strategy of rollback satisfies the correctness criteria of hybrid checkpointing model.

**Proof.**  By contradiction. If the rollback strategy does not satisfy the correctness criteria, then there must be a message *M* there must be a message *M* whose sender process has cancelled the sending event of *M* but the receiver process does not cancel the receiving event of *M*. Let *P_i_* and *P_j_* to be the sender process and receiver process of *M* respectively. *CKP_i_* and *CKP_j_* are the newest checkpoints of *P_i_* and *P_j_* when they send and receives *M*, respectively.

1. *M* is a *SM*. By the time *P_j_* receives *M*, the IntraCDG_j_ of *P_j_* will record the dependence relation of *CKP_j_* ⇒ *CKP_i_*. If some process in the cluster same as *P_j_* build the IntraCDGglobal for failure, then there must be a path from CKP_i_ to *CKP_j_*. Therefore, *P_j_* is sure to roll back to *CKP_j_* because *CKP_j_* belongs to *RS_j_* when *P_i_* rolls back to *CKP_i_* for failure or rollback request.
2. *M* is a DM. At present, *CKP_j_* is *DC_j_.* After *Pi* sends *M*, the cluster head *CH_k_* of cluster same as *P_i_* will firstly receive *M*. When CH_k_ receives M, it transmits M and records the dependence relation of *CKP_j_* ⇒ *CKP_i_* in S_InterCDG_k_. When *Pi* rolls back to *CKP_i_* for failure or rollback request, *CH_k_* is sure to require P_j_ to roll back to *CKP_j_* because there is edge started by *CKP_i_* and ended by *CKP_j_* in S_InterCDGk.

So, it follows that the assumption for rollback strategy does not set up. The rollback strategy meets the correctness criteria.

**Algorithm 4 CKP_Rollback( )****for** (*P_j_* ∈ *PL_k_* ∧ *j* ≠ *i*) **do** SEND_IntraCDGReq(*P_j_*); //send *IntraCDG* request BUILD_IntraCDG(*IntraCDGglobal*); Traverse(*IntraCDGglobal*, *CKP_i_*); //traverse *IntraCDGglobal* by depth-first **for** (*P_j_* ∈ *PL_k_* ∧ *j* ≠ *i*) **do** SEND_ERS(*P_j_*); **for** (*P_j_* ∈ *PL_k_*) **do** **if** (*CKP_s_* ∈ *ERS_i_* ∧ *CKP_s_* ∈ *P_j_*) **then** Rollback(*P_j_*, *CKP_s_*); **for** (*CKP_s_* ∈ *ERS_i_* ∧ *CKP_s_* ∈ *S_InterCDG_k_*) **do** **if** (∃Edge(*CKP_s_*, *DC_j_*)) **then** SEND_RollReq(*DC_j_*); //send rollback request to process related to *DC_j_*

## 7. Processing of Handoffs

A MH leaves one cluster and enters another cluster in the ad hoc network, namely a handoff occurs, then the relations of the MH in same cluster and different clusters all change. We call this MH that passes the handoff as the HandOff Host (HOH). The cluster that HOH leaves is called old cluster and the cluster that HOH enters is called new cluster.

After the handoff has happened, there will be some situations. If the old cluster does not deal with this change in time, then any checkpointing procedure of processes in old cluster may fail to accomplish because of the absence of HOH. The rollback of processes may also not be realized, because the processes could not obtain all of dependence relations of checkpoints. If the new cluster does not deal with this change in time, then the *DCs* of HOH may not be able to be cleared away and affects the elimination of more checkpoints further. What is more, the checkpointing in new cluster will ignore the entering of HOH and so cause the incoordination of checkpoints. Hence, the HOH, old cluster and new cluster all must deal with this change relatively for assuring the correctness of checkpoints when the handoff happens.

### 7.1. Process States and Treatment

**Figure 8:**
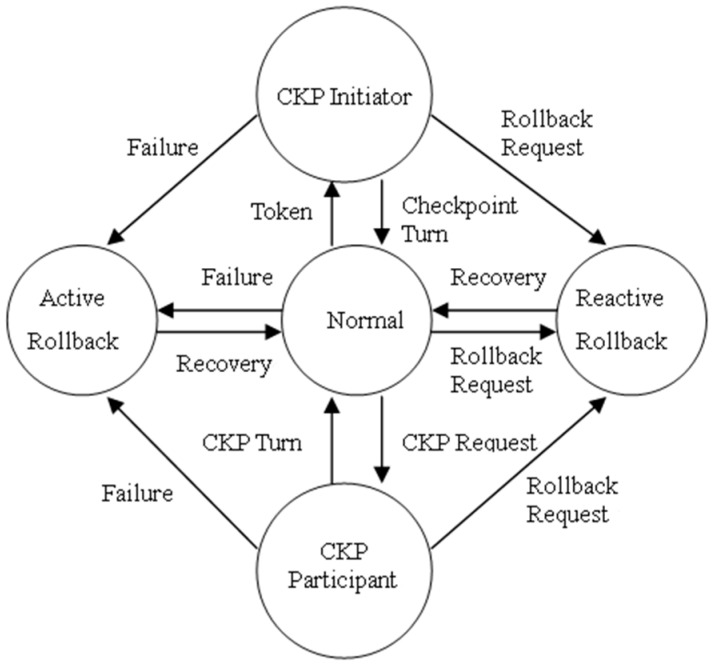
The transition of states.

For the processes running in the HOH, they stay one of the following states when the handoff happens. Figure 8 is the transition figure of states.

1. Running state. The process runs or communicates normally.
2. Checkpoint state. The process is taking checkpoint. The process can be divided to *CKP* Initiator or *CKP* Participant. The *CKP* Intiator means that the checkpointing procedure is initialized by this process, while the *CKP* Participant means that this process just take part in the checkpointing procedure initialized by other process.
3. Rollback state. The process is just dealing with the rollback recovery. This state can be divided to Active Rollback and Reactive Rollback state. The Active Rollback state indicates that the process is the failure process, namely the initiator of rollback. While the Reactive Rollback state means that the process rolls back because of the cascade rollback caused by rollback request of another process.

When the HOH leaves the old cluster, and enters the new cluster, the processes running in HOH should be able to handle according to their different states. Despite the processes staying normal state, all processes running in HOH have to finish the current work (checkpointing or rollback operation) with the processes in old cluster together for assuring the consistency of checkpoints. Hence, the HOH informs its location and state to the *CH* of old cluster when it enters the new cluster. Then the *CH* judges whether it is needful to notice the processes in its cluster.

#### 7.1.1. The Process Stays in the Rollback State

Active Rollback. The HOH goes on obtaining the *IntraCDG* of processes in the old cluster and calculates the *ERS* according to *IntraCDG*. The *LCH* sends the cluster *id* of new cluster to the CH of old cluster. Then the process executes rollback operation according to *ERS* and sends the *ERS* to related processes in old cluster to inform them to roll back.

Reactive Rollback. The process waits for the *ERS* sent by the process running in old cluster and executes rollback after received the *ERS*.

#### 7.1.2. The Process Stays the Checkpoint State

*CKP* Initiator. If the process stays the receiving stage of DL, then it goes on waiting for the DL sent from processes in old cluster. If the process has sent the *LDS*, then it waits for the replies from processes in old cluster. After that, the process continues executing the checkpointing until the end.

*CKP* Participant. The process waits for the coordination request from the initiator of checkpointing and replies until the end of checkpointing procedure.

### 7.2. Update of Checkpoint Dependence Relation

Because the the relations of the HOH in same cluster and different clusters all change after it leaves the old cluster and enters the new cluster, so it needs to update the dependence relations among checkpoints in old or new cluster and the checkpoints of processes in HOH when the all processes in HOH have finished their current work. The main update work is to renew the related data structures such as the *PL*, *DL*, *IntraCDG* and *InterCDG*. Let *C_k_* and *C_i_* respectively the old cluster and new cluster related to HOH. *PS* is the set of processes running in HOH.

#### 7.2.1. Update of Checkpoints Dependence Relation in Old Cluster

Firstly, it needs to delete the processes in *PS* from the *PL_k_* and *DL_s_* of all processes in old cluster. Then it should turn the same cluster dependence relation among checkpoints of processes in *PL_k_* and *PS* to dependence relations among different clusters. That means to modify the related *IntraCDG* and *InterCDG*.

Let *P_t_* ∈ *PS*, *P_j_* ∈ *PL_k_*. *CKP_a_* and *CKP_b_* are the checkpoints of *P_t_* and *P_j_* respectively. The algorithm for updating of checkpoints dependence relations in old cluster is described as the Algorithm 5.

**Algorithm 5 OldCluster_CKPUpdate( )** DELETE_process(*PL_k_*, *PS*); //delete *PS* from *PL_k_* NOTICE_DLChange(*PS*); //notice processes to change *DL* **for** (*P_x_* ∈ *PS* || *P_x_* ∈ *PL_k_*) **do** REQUIRE_IntraCDG(*P_x_*); //send *IntraCDG* request BUILD_IntraCDG(*IntraCDGglobal*); **if** (∃Edge(*CKP_a_*, *CKP_b_*) ∧ *IntraCDGglobal*) **then** CHANGE_IntraCDG(*IntraCDG_j_*); //modify *IntraCDG_j_* CHANGE_InterCDG(*R_InterCDG_k_*); **if** (∃Edge(*CKP_b_*, *CKP_a_*) ∧ *IntraCDGglobal*) **then** CHANGE_IntraCDG(*IntraCDG_t_*); CHANGE_InterCDG(*S_InterCDG_k_*); BUILD_SubInterCDG(*R_InterCDG_k_*, *S_InterCDG_k_, P_t_*); //build subgraph related to *P_t_* SEND_SubInterCDG(*CH_i_*); //send subgraph to *CH_i_* CHANGE_InterCDG(*R_InterCDG_k_*); CHANGE_InterCDG(*S_InterCDG_k_*);

#### 7.2.2. Update of Checkpoints Dependence Relation in New Cluster

Firstly, it needs to add the processes in *PS* to *PL_i_.* When *CH_i_* receives the subgraph *SubInterCDG* from *CH_k_*, it adds the edges ended by the checkpoints of processes in *PS* to *R_InterCDG_i_* and adds the rest to *S_InterCDG_i_*. Then *CH_i_* builds new relations of same cluster for the processes in *PS*, namely updates the *IntraCDG_s_* and *DL_s_* of related checkpoints.

Let *P_t_* ∈ *PS*, *P_s_* ∈ *C_i_*. *CKP_c_* and *CKP_d_* are the checkpoints of *P_t_* and *P_s_*, respectively. The algorithm for updating checkpoints dependence relations in a new cluster is described as the Algorithm 6.

**Algorithm 6 NewCluster_CKPUpdate( )** ADD_process(*PL_i_*, *PS*); //add *PS* to *PL_i_* CHANGE_InterCDG(*SubInterCDG*, *R_InterCDG_i_)*;  //modify *R_InterCDG_i_* with *SubInterCDG* CHANGE_InterCDG(*SubInterCDG*, *S_InterCDG_i_*); **if** (∃Edge(*CKP_c_*, *CKP_d_*) ∈ *R_InterCDG_i_* || ∃Edge(*CKP_d_*, *CKP_c_*) ∈ *S_InterCDG_i_*) **then** CHANGE_dependence(*CKP_c_, CKP_d_*);  //change dependence relation of *CKP_c_* and *CKP_d_* **for** (*P_x_* ∈ *PL_i_*) **do** NOTICE_IntraCDGchange(*P_x_*); **for** (*CH_m_*, *m* ∈ *k* ∧ *m* ∈ *i*) **do** NOTICE_clusterchange(*CH_m_*, *P_t_*); //notice *CH_m_* the cluster change of *P_t_* **for** (*P_x_* ∈ *PL_i_*) **do** CHANGE_IntraCDG(*P_x_*); //*P_x_* modify *IntraCDG* CHANGE_DL(*P_x_*); //*P_x_* modify *DL*

## 8. Performance Test and Analysis

### 8.1. Experiment Environment and Parameters

This experiment builds a simulation environment of a mobile ad hoc network based on Windows XP. The simulation network consists of five clusters. Each cluster includes 5 MHs. The routing protocol in the experiment is the Ad hoc On-Demand Distance Vector Routing (AODV). The size of one checkpoint and one message log are set to 2 KB and 50 B, respectively. Table 1 shows the parameters of this experiment.

This experiment plans to compare the proposed hybrid checkpointing protocol (HCP) with the pure Synchronous Checkpointing Protocol (SCP) and the pure Asynchronous Checkpointing Protocol (ACP). In the pure synchronous checkpointing strategy, a process sends the coordination request to the related processes in same cluster and different clusters after received *K* messages. The process firstly takes an uncertain checkpoint, and then turns the uncertain one to certain one after the coordination is successful. In the pure asynchronous checkpointing strategy, a process takes a checkpoint independently after received *K* messages. According to the characteristics of ad hoc networks, this experiment adopts the following performance indexes:

1. Average Checkpoint Time (ACT): average time for finishing the checkpointing procedure;
2. Average Recovery Time (ART): average time for finishing the rollback recovery operation;
3. Average Storage Size (ASS): average disk space that is occupied by the checkpoints of *MH*;
4. Average Additional Messages (AAM): average number of messages for checkpoints coordination and rollback recovery despite the normal communication messages;
5. Average Cluster head Forwarded Messages (ACFM): average number of messages forwarded by the cluster head.

### 8.2. Experiment Results and Analysis

This experiment divides to two parts. The first part is to test the message threshold for checkpoint *K* in the experiment environment. The second part is to test the *ACT*, *ART*, *ASS*, *AAM* and *ACFM* of the three protocols under different conditions.

#### 8.2.1. Experiment 1 Selection of *K*

The checkpoint frequency of a process is decided by the message threshold *K* in HCP. If *K* enlarges, then the number of checkpoints deceases and the *ART* increases. What is more, if *K* increases, then the logs between checkpoints increase. Because the storage overhead of checkpoints is much larger than the average storage overhead of message logs, so the storage overhead will reduce as *K* increases.

**Figure 9:**
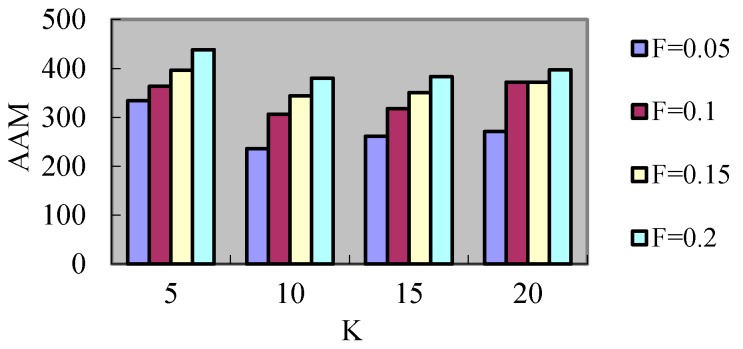
Test for a suitable value of K.

For the additional messages, the checkpoint frequency deceases as *K* increases and so the additional messages for checkpoint coordination reduce, but the additional messages for rollback recovery augment, so the *AAM* does not maintain an absolute downtrend as *K* augments. Hence, this experiment takes the lower of *AAM* as the main parameter for selection of *K*. Figure 9 shows the *AAM* for varying values of *K* under different failure probabilities. It can be seen that the *AAM* under different failure probabilities is the least when *K* is 10. Therefore, the follow-up experiments choose this value of *K* to analyze and compare.

#### 8.2.2. Experiment 2 Test of *ACT*

**Figure 10:**
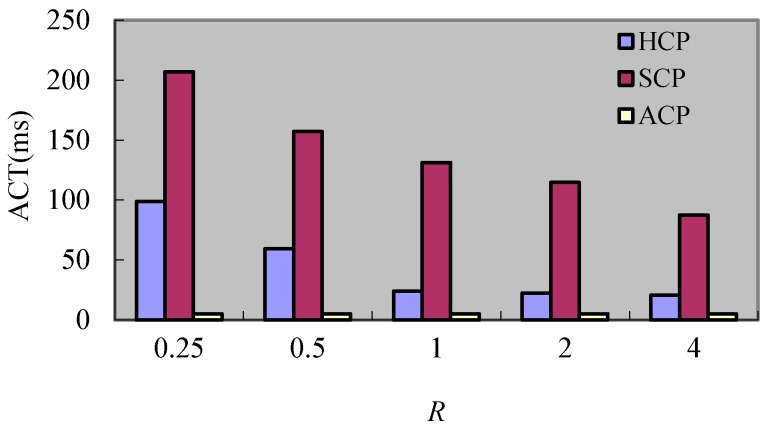
Impact of R on ACT.

Figure 10 shows the *ACT* for varying ratios of SM number to DM number (*R*). It can be seen that because the checkpoints in ACP do not remain coordinated with other processes and the processes just need to store the current states of processes, so the *ACT* does not change at all. However, the *ACT* of HCP and SCP reduces as *R* augments. For the HCP, the number of processes that participate in the coordination decreases when the processes satisfy the checkpoint condition as *R* increases, so the *ACT* reduces as *R* augments. For the SCP, the number of DM_s_ diminishes as *R* increases and it means that the processes in different clusters which also have to join the coordination reduction. Hence, the *ACT* decreases as *R* augments. What is more, the time for checkpoints coordination of HCP is sure to be less than the SCP, because the checkpoints coordination of HCP is limited in one cluster.

#### 8.2.3. Experiment 3 Test of *ART*

**Figure 11:**
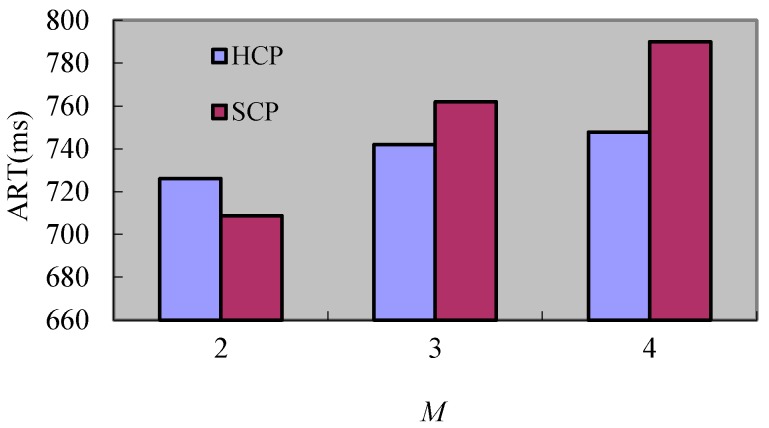
Impact of M on ART.

**Figure 12:**
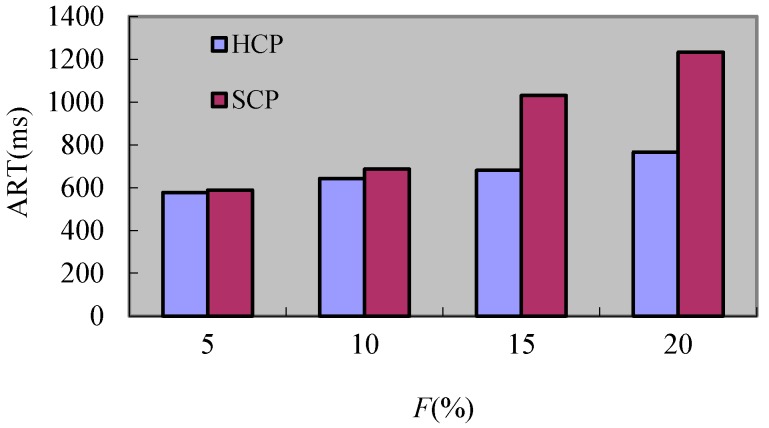
Impact of F on ART.

Because there are major cascade rollbacks in ACP, so the *ART* of ACP is greatly large than the one of HCP and SCP. In order to clearly describe the difference between HCP and SCP, this experiment just compares the effect on *ART* of HCP and ACP for varying *M* and *F*. The results are displayed in Figure 11 and Figure 12.

As Figure 11 shows, the *ART* of SCP is lower than the one of HCP when *M* is 2. This is because the extent of change of the rollback caused by the cascade rollback of HCP is larger than the one brought by the rollback request of SCP, but the *ART* of these two protocols all increase as *M* augments and the time of HCP is lower than SCP when *M* is 3 and *M* is 4. The reason for the augment of two algorithms is that there are rollback requests sent to different clusters. Thus, the rollback extent increases when processes in different clusters receive the rollback requests as *M* augments. By contrast, the rollback requests sent to different clusters of SCP are greatly larger than the ones of HCP because the rollback of SCP is global. Hence, the rollback extent change of SCP caused by *M* is greater than the rollback extent change of HCP brought by the cascade rollback when *M* is larger.

It can be seen from Figure 12, the recovery time of HCP and SCP all increase when *F* augments but the recovery time of HCP is less than the one of SCP. This is because the communications between the checkpoint and failure point decrease and then the related rollback processes reduce. Hence, the rollback extents of processes that do not fail have larger rollback extents when they roll back next time. This situation causes the increment of recovery time. Because the rollback notifications sent to different clusters of HCP is lesser, so the *ART* of HCP is less than the one of SCP.

#### 8.2.4. Experiment 4 Test of *ASS*

**Figure 13:**
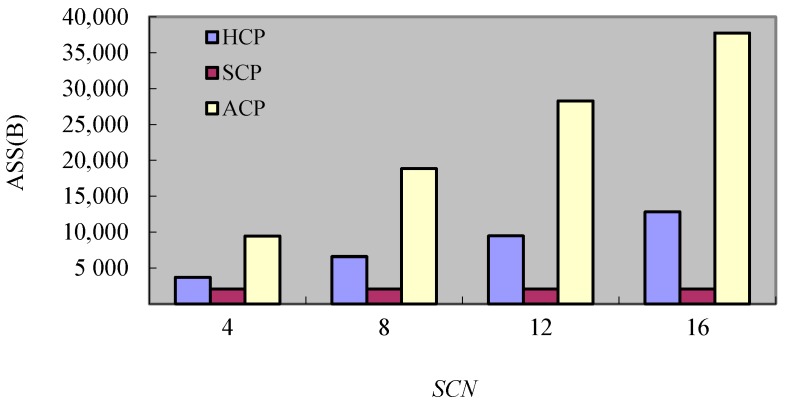
Impact of SCN on ASS.

In order to test the *ASS*, this experiment compares the occupation of storage space of different protocols by testing the change of *SCN*. Figure 13 shows the results of tests. It can be seen, the *ASS* of SCP does not change basically. This is because each process in SCP just needs to store one checkpoint and a handful of message logs. However, each process in HCP and ACP has to store multiple checkpoints and many message logs, and so the occupation of storage space increases as the successive checkpoints number augments. But the *ASS* of HCP is less than the *ASS* of ACP greatly because the *DC_s_* of HCP will be cleared away in advance.

#### 8.2.5. Experiment 5 Test of *AAM*

**Figure 14:**
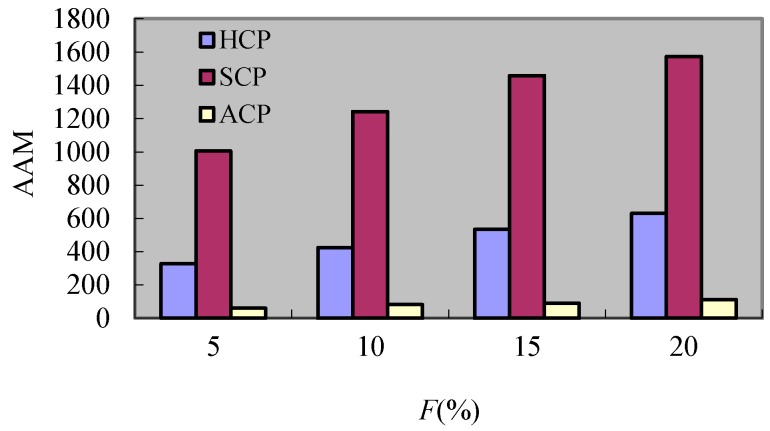
Impact of F on AAM.

**Figure 15:**
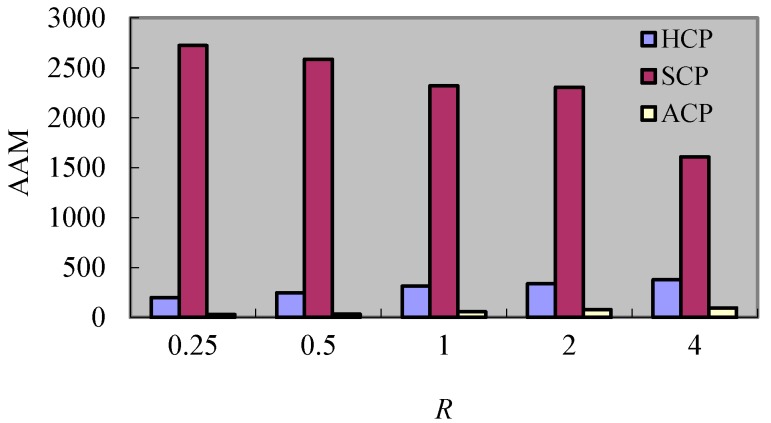
Impact of R on AAM.

In order to test the *AAM*, this experiment compares the effect of change created by *F* and *R* respectively. The performance results of this experiment are described in Figure 14 and Figure 15. It can be seen from Figure 14 that the additional messages increase along with the increment of *F*. This is because the larger the *F* is, the more additional messages related to rollbacks exist, but only the *AAM* of SCP shows a downtrend as *R* augments. The reason is that the processes which participate in the coordination decrease with the reduction of number of *DM_s_* when *R* increases, but for the HCP and ACP, the increment of *R* causes the dependence relations among checkpoints of processes to become tighter and so the additional messages created by the rollbacks of failure processes augment. In general, the *AAM* of SCP is greatly larger than the one of HCP and ACP. This is because the SCP has the global rollback despite the global coordination, but for the HCP, though there are additional messages caused by coordination and rollback together, the coordination and rollback are limited in the same cluster. Hence, the additional messages of HCP are less than S for CP. For the ACP, its additional messages are the least because there is no coordination in ACP.

#### 8.2.6. Experiment 6 Test of *ACFM*

**Figure 16:**
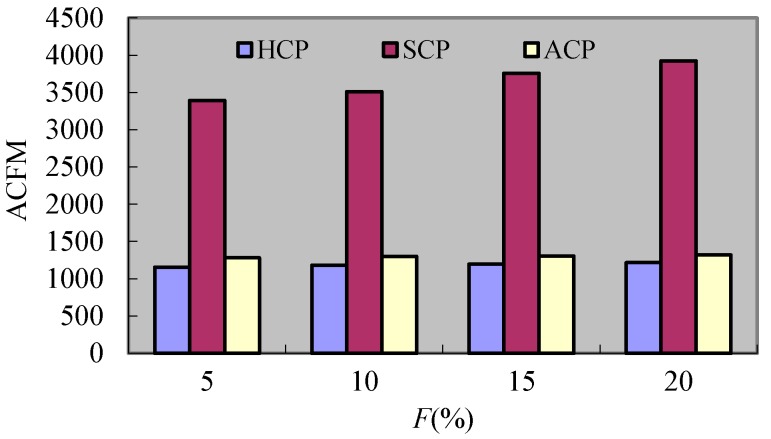
Impact of F on ACFM.

**Figure 17:**
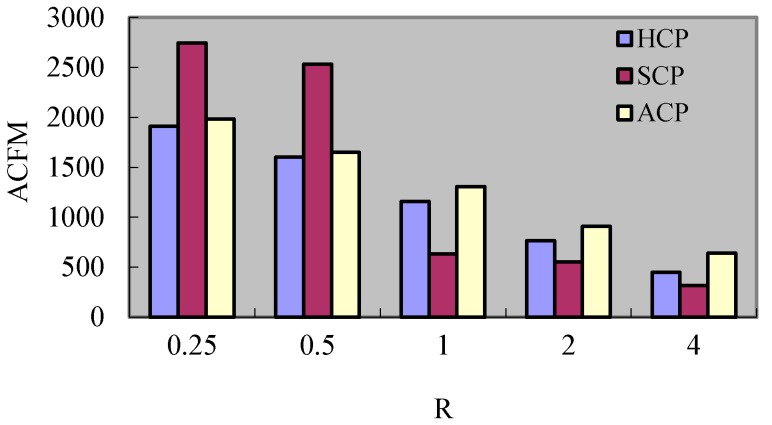
Impact of R on ACFM.

Figure 16 and Figure 17 express the change of *ACFM* for the three protocols under different *F* and *R*. It can be seen that the enlargement of F causes the increment of *ACFM* in the three protocols. Hereinto, the change extents of HCP and ACP are lesser. This is because the rollback requests forwarded by *CH_s_* increase when *F* augments. For the HCP and ACP, the rollback requests forwarded by *CH_s_* are lesser, and so is the effect of *F*. In the Scp, each rollback requires all processes to partake and so the messages forwarded by *CH_s_* become major and take up a high proportion of the total messages. Then it causes the change extent of messages forwarded by *CH_s_* being larger when *R* varies. What is more, the CH_s_ in SCP will forward the coordination requests also and so the messages forwarded by *CH_s_* in Scp are the largest. For the HCP, the coordination requests for different clusters and rollback requests forwarded by *CH_s_* are lesser, and so the messages forwarded by *CH_s_* are less than ACP. As *R* increases, the *ACFM* of the three protocols all reduce. Hereinto, the *ACFM* of HCP is least when *R* is less than 1. This is because the kinds of requests related to processes in different clusters increase when *R* augments. So the messages forwarded by *CH_s_* reduce. For the SCP, the related coordination processes in different clusters decreases with the increment of *R* when *R* is larger than 1, and so the coordination requests forwarded by *CH_s_* reduce obviously. In the HCP, though the coordination and rollback requests for different clusters decrease with the increment of *R*, there are a certain amount of elimination notices of *DC_s_*. Hence, the reduction extent of HCP is less than the one of SCP.

In short, because each process in SCP just stores one checkpoint, so the occupation of storage space is lesser. There is no cascade rollback in SCP, thus the recovery time is short, but in order to assure the checkpoints coordination of all processes, the processes in SCP need to exchange a lot of coordination information and so the additional messages are major.

In the ACP, because the checkpoints of processes are not synchronous, so the checkpoint time is just the time needed to store the state of the process and the additional messages just include bits of additional messages caused by rollback. However, the storage space of ACP is larger because of the storing of many checkpoints. What is more, the recovery time is longer, because there are cascade rollbacks among checkpoints of processes.

For the proposed hybrid protocol HCP, the time to transmit and receive the kinds of coordination information is less than the one of SCP, because the coordination of checkpoints is limited to the processes in same cluster. Hence, the checkpoint time of HCP is greatly shorter than the one of SCP. In addition, the coordination and rollback of HCP do not affect the other clusters marginally and so the retransmission overhead of *CH_s_* is lesser. Though the additional messages of HCP are a little higher than the ones of ACP because of coordination, the recovery time of HCP is shorter for bits of constraints from different clusters and cascade rollbacks.

## 9. Conclusions and Next Work

Mobile ad hoc networks are an important part of modern communication networks, but the existing checkpointing algorithms could not be applied to this network environment very well because it has the features such as less of support of fixed network, multi-hops routing, changeable topological structure, bad stability and high failure probability.

In the distributed system, the performance indexes for measuring checkpoints mainly include the number of communication messages, storage overhead, recovery overhead and time for checkpointing coordination, so it can choose between coordinated or uncoordinated checkpointing according to the characteristics of application environment. However, there is no support for fixed networks and the kinds of resources are very limited in mobile ad hoc networks. For example, the wireless bandwidth is lower and the CPU capability is weak in mobile ad hoc networks, so the checkpointing technologies for this kind of network cannot only consider any index alone, and a trade off method which takes kinds of performance indexes (such as checkpoint time, rollback recovery time, storage space and number of additional messages) into account synthetically should be designed. Therefore, this paper designs a hybrid checkpointing model and algorithm which combines the synchronization with asynchronization technology. This hybrid checkpointing algorithm not only avoids the major cascade rollback among MH_s_ in the same cluster and too much message transmissions among the MH_s_ in different clusters, but also reduces communication delay. What is more, this hybrid model has little dependence on CH_s_, so it has good flexibility.

Compared to the pure synchronous and asynchronous checkpointing strategy, this paper does plenty of performance tests for the hybrid checkpointing strategy. The results show that the hybrid checkpointing strategy is a good trade off method between the synchronous and asynchronous checkpointing strategy. What is more, it has shorter recovery time compared with the two methods.

Our next work is to analyze the multicast communication protocols and kinds of routing protocols and then design a new checkpointing algorithm which is suitable to the multicast environment and adapts to the different routing protocols. To do so, the fault tolerance and reliability of ad hoc networks will be further improved.

## Figures and Tables

**Table 1:** Parameters for Experiments.

Parameter	Description	Value
*F*	Failure probability	5~20%
*M*	Average hops between MHs	2~4
*K*	Message threshold for checkpoint	10
*SCN*	Successive checkpoints number	4~16
*R*	Ratio of SM number to DM number	0.25~4
